# Micro-Displacement Sensor Based on a Hollow-Core Photonic Crystal Fiber

**DOI:** 10.3390/s121217497

**Published:** 2012-12-17

**Authors:** Ana Margarida Rodrigues Pinto, José Manuel Baptista, José Luís Santos, Manuel Lopez-Amo, Orlando Frazão

**Affiliations:** 1 Departamento de Ingeniería Eléctrica y Electrónica, Universidad Pública de Navarra, Campus de Arrosadía, 31006 Pamplona-Navarra, Spain; E-Mail: mla@unavarra.es; 2 Instituto de Engenharia de Sistemas e Computadores do Porto (INESC Porto), Departamento de Física, Faculdade de Ciências, Universidade do Porto, Rua do Campo Alegre, 687, 4169-007 Porto, Portugal; E-Mails: jmb@uma.pt (J.M.B.); josantos@fc.up.pt (J.L.S.); ofrazao@fc.up.pt (O.F.)

**Keywords:** fiber optic sensor, photonic crystal fiber, micro-displacement sensor, modal interferometer

## Abstract

A sensing head based on a hollow-core photonic crystal fiber for in-reflection measurement of micro-displacements is presented. The sensing structure takes advantage of the multimodal behavior of a short segment of hollow-core photonic crystal fiber in-reflection, being spliced to a single mode fiber at its other end. A modal interferometer is obtained when the sensing head is close to a mirror, through which displacement is measured.

## 1. Introduction

Since the development of the first band gap guiding photonic crystal fiber (PCF) in 1999, a lot of attention has been paid to hollow-core photonic crystal fibers (HC-PCF). HC-PCF geometry is characterized by a microstructured air hole cladding running along the entire length of the fiber surrounding a hollow core. Light guiding is accomplished in these fibers through photonic bandgap instead of total internal reflection (TIR), making possible to escape from the straitjacket geometry needed to obtain TIR and making possible the trapping of light in an air hollow-core. Furthermore, these fibers present remarkable advantages such as extremely small Fresnel reflections with the outside world due to small refractive index discontinuity, low interaction between the guided light and the material forming the fiber and the ability to fill the core of the fiber with gases and liquids leading to high light/sample overlapping [1,2]. These advantages lead to fascinating applications based on HC-PCFs, such as gas sensing [3], rhodamine sensing [4], DNA sensing [5], particle guidance and levitation [6], among others. However, hollow-core PCFs present the disadvantage of supporting multiple modes: besides the fundamental core mode they present higher order core modes, cladding modes and surface modes [7]. Even so, there is substantial attenuation of the different types of modes along the fiber length and after light propagation through a long length of the fiber only core guided modes subsist. Nevertheless, this disadvantage related with small lengths of HC-PCFs can be used for practical purposes. The multi-modal operation at short lengths of HC-PCFs opens up the possibility to build modal interferometers, with potentially interesting characteristics for optical fiber sensing. A modal interferometric structure based on a piece of HC-PCF connected in both ends to standard single mode fiber (SMF) was accomplished demonstrating sensitivity to strain and temperature [8].

Monitoring of displacement changes is of practical interest for areas such as aeronautics, metallurgy, and health monitoring of complex structures, among others. With this purpose, several types of displacement sensors were developed based on fiber-optic techniques due to the known advantages of this technology, for instance, immunity to electromagnetic interference, low weight, remote sensing ability, and high multiplexing capability. To this date, a number of displacement fiber sensors using PCFs based on interferometry have been reported: a modal interferometer was obtained through a structure composed by SMF-PCF-SMF with a core offset at one of the joints presenting a sensitivity of 0.0024 dB/μm to displacement changes [9]; using a Hi-Bi PCF in a Sagnac interferometer a displacement sensor was reported with a sensitivity of 0.28286 nm/mm [10]; and through a three-hole suspended-core fiber Sagnac loop mirror, a displacement sensor was developed with high precision (∼0.45 μm) [11].

In this paper, a study of a hollow-core photonic crystal fiber based in-reflection interferometer for micro-displacement measurement is presented. The sensing structure is obtained through splicing a piece of hollow-core photonic crystal fiber to a single mode fiber, leaving its other end cleaved. An interferometer is obtained when the sensing head approaches a mirror, through which displacement is measured.

## 2. Experimental Setup and Results

**Figure 1.**
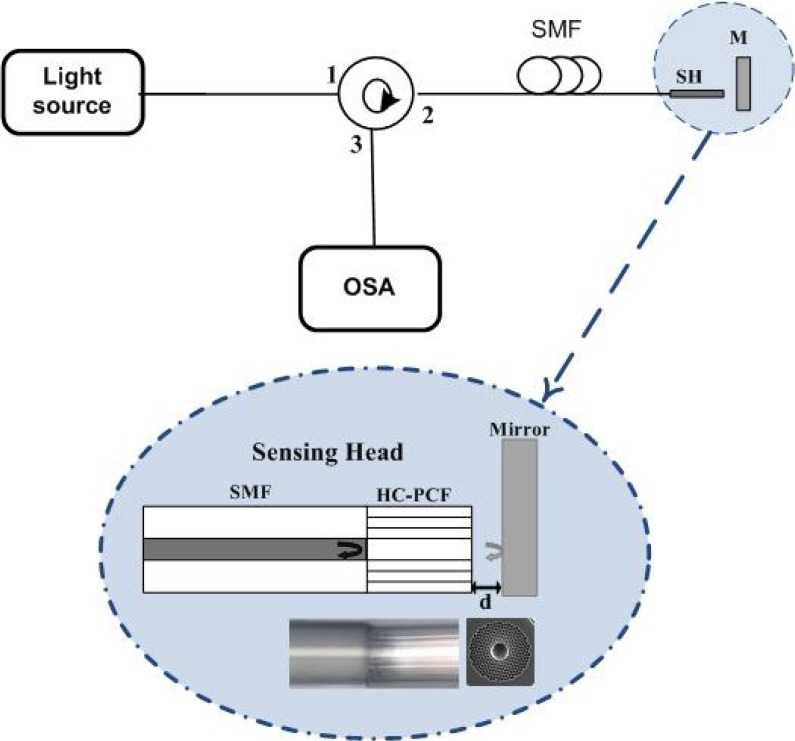
Schematic diagram of the set up used to measure displacement using the sensing head. Inset: drawing of the sensing head, below microscope photo of the splice zone in the sensing head, and at the right microscope photo of the cross section of the HC-PCF.

The schematic configuration and the sensing head used for displacement measurement are shown in Figure 1. The set up consists of a broadband light source illuminating the sensing head through a circulator. The signal was observed using an optical spectrum analyzer (OSA) with a maximum resolution of 10 pm. The sensing head was fabricated through splicing one end of a piece of HC-PCF to a SMF while leaving the other side cleaved—a photo of the splice zone is shown on the inset of Figure 1. The hollow-core PCF (HC19-1550 from NKT Photonics) was obtained by removing 19 cells from the cladding presenting a core diameter of 20 μm, 115 μm of diameter, a pitch of 3.9 μm and an air filling fraction higher than 90%, resulting in a multimodal fiber with ∼30 modes over a few centimeters. A photo of the cross section of this HC-PCF can be also seen on the inset of Figure 1.

**Figure 2.**
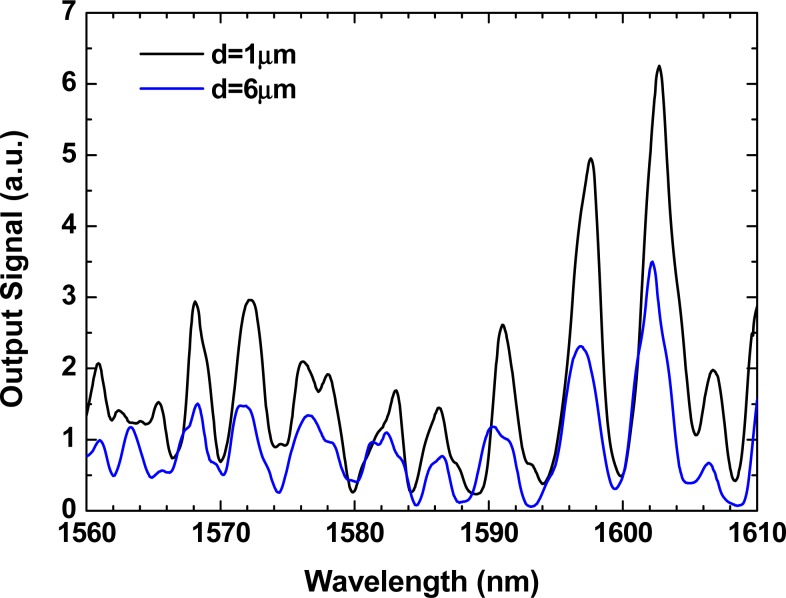
Sensing head interferometric signal output, for a cavity length of 2.5 cm and two different distances.

As can be seen from the inset of Figure 1, this sensing head presents a reflection in the interface between the SMF and the HC-PCF. In addition, for short lengths, HC-PCFs present multiple propagation modes (fundamental core mode, higher order core modes, cladding modes and surface modes). Moreover, when approaching the sensing head to the mirror, part of the modes guided in the short length of HC-PCF are reflected and coupled back into the HC-PCF. Consequently, a modal interferometer is obtained when approaching the sensing head to the mirror. This modal interferometer output will regularly change with the micro-displacement between the sensing head and the mirror, allowing in this way a correct measurement of displacement variations. Thus, to measure the output signal variation with displacement, the mirror was pulled apart in steps of 1 μm, with an estimated error of 0.5 μm. Figure 2 presents the modal interferometer obtained with a HC-PCF of 2.5 cm of length for two different displacements. It was experimentally observed that the sensing head output signal variations with input polarization changes were negligible. Even more, an experimental verification was made of the sensing head sensitivity to temperature changes. It was observed that the sensing head as negligible fluctuations of power with temperature changes, as it was expected since only a small fraction of light propagates in glass in the hollow-core photonic crystal fiber (<3%).

**Figure 3.**
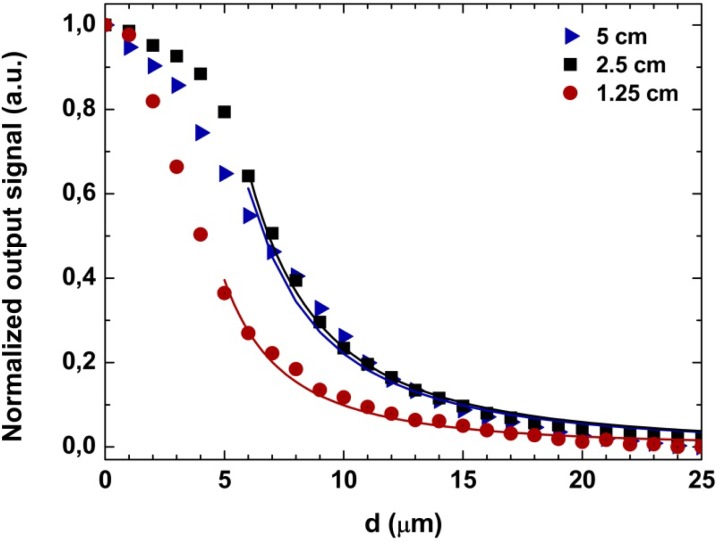
Intensity response of the sensor with displacement changes, for three different cavity lengths.

The experimental intensity response of the sensing head with displacement is presented in Figure 3 as dots, squares and triangles. As it can be seen, by monitoring the alterations of a fringe in the interferometric spectrum when displacement changes are imposed to the sensing head, the intensity presents a non-lineal variation as a function of the distance between the sensing head and the mirror.

**Figure 4.**
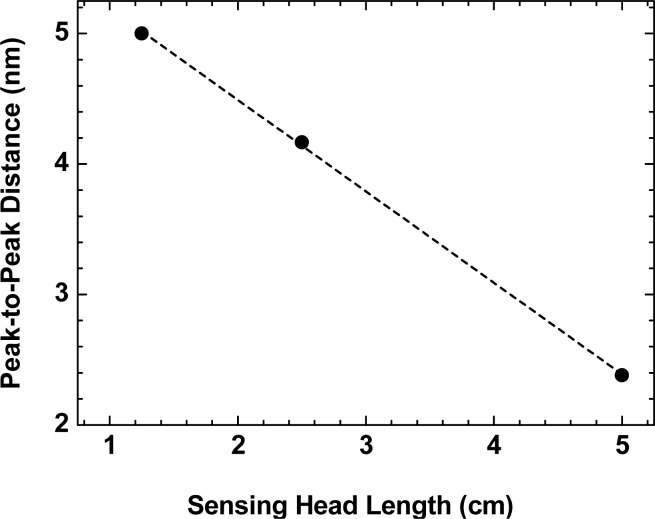
Experimental variation of the peak-to-peak distance of the fringes of the interferometric output with the sensing head length.

The response of the intensity with the displacement varies in accordance with the inverse square law accordingly with the equation:

$$\frac{P_{\mathrm{out}}}{P_{\mathrm{in}}}=\frac{r^{2}}{{\left(2d\;\text{tan}\theta \right)}^{2}}=\frac{k}{d^{2}}$$

where *P_out_* is the output power, *P_in_* in the input power, *r* is the fiber radius, *d* is the displacement, *θ* = *sin*^−1^(*NA*) and *k* is a constant. This theoretical variation in represented in Figure 3 by the blue, black and red lines for the 5 cm, 2.5 cm and 1.25 cm sensing heads, respectively. It can be observed from Figure 3 that theoretical and experimental values are quite in agreement, apart from the few first micrometers of displacement. This behavior is similar to the conventional micro-displacement based on the standard fiber combined with a external mirror [12]. For the few first micrometers a non-linear behavior is obtained. This is due to the fact that the instrumentation used for the alignment of the sensing head and the mirror did not allow us to ensure that they were completely parallel to each other, and a small angle was formed between, causing a misalignment which in turn causes this erratic behavior over short distances, becoming negligible at higher displacements. In order to enhance the performance of the sensor, specific signal processing techniques for interferometric sensors can also be used [13]. In the linear region of the data presented in Figure 3, the sensitivities obtained to displacement are 0.22 μW/μm for the 5 cm sensing head, 0.685 μW/μm for the 2.5 cm sensing head and 0.727 μW/μm for the 1.25 cm sensing head. These sensitivities are two orders of magnitude higher than a fiber optic displacement sensor based on a multimode plastic 50:50 coupler shown to have a sensitivity of 0.00628 μW/μm [12]. Another conclusion that can be taken from the obtained sensitivities is that the smaller the sensing head length the higher its sensitivity is. By studying the sensing heads’ characteristics, it was also observed that the wavelength spacing between the fringes of the interferometric spectrum decays linearly with higher lengths of the sensing head, as depicted in Figure 4. As can be seen, for shorter sensing head lengths the modal interferometer presents higher peak-to-peak distances. This entails that the sensing head can be tailored for any intended application by properly choosing its length.

**Figure 5.**
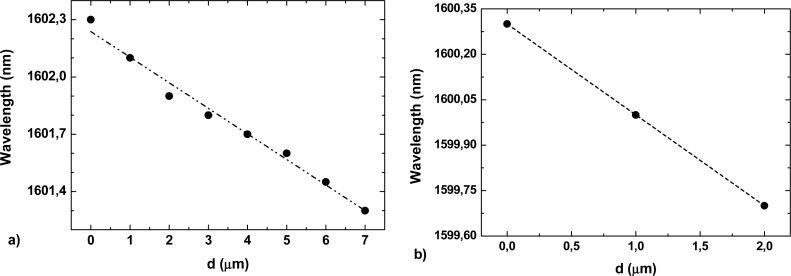
Wavelength response of the sensor for displacement changes, for sensor cavity length of (**a**) 2.5 cm and (**b**) 1.25 cm.

A study of the wavelength response with displacement was done for each sensing head. Figure 5 presents the wavelength response obtained by monitoring the shift of a fringe positioned at 1,602.3 nm and 1,600.3 nm in the interferometric spectrum for the 2.5 cm and 1.25 cm sensing heads, respectively. The interferometric fringes were linearly shifted toward shorter wavelengths with the increase of displacement, presenting sensing slopes of −0.134 nm/mm and −0.3 nm/mm for the 2.5 cm and 1.25 cm sensing heads, respectively. The sensing head with 5 cm did not present a shift in wavelength with displacement.

## 3. Conclusions

A study of a hollow-core photonic crystal fiber in-reflection sensing head for displacement measurement was proposed and demonstrated. This is the first time, to the best of our knowledge, that a hollow-core photonic crystal fiber is used as a displacement sensor in reflection. The sensing structure is obtained by taking advantage of the multimode behavior of a short piece of hollow-core photonic crystal fiber in reflection. The resulting modal interferometer was used to measure displacement between the sensing head and a mirror. The intensity response presented an inverse square distance response with displacement changes, presenting different sensitivities depending on the length of the sensing head. The sensitivities obtained were 0.22 μW/μm, 0.685 μW/μm and 0.727 μW/μm for the 5 cm, 2.5 cm and 1.25 cm sensing heads, respectively. The sensing heads present negligible power fluctuations to polarization changes. Furthermore, since only a small fraction of the light propagates in glass in the hollow-core photonic crystal fiber (<3%) this sensing head presents negligible fluctuation of power output with temperature variations. The simple and compact design of this sensing head allied with the presented performances provide a very attractive solution for applications such as micro-displacement measurements in hazardous environments, position control and monitoring of automated control.

## References

[1] Russell P.S.J. (2006). Photonic-crystal fibers. J. Lightwave Technol.

[2] Pinto A.M.R., Lopez-Amo M (2012). Photonic crystal fibers for sensing applications. J. Sens..

[3] Cubillas A.M., Lazaro J.M., Silva-Lopez M., Conde O.M., Petrovich M.N., Lopez-Higuera J.M. (2008). Methane sensing at 1,300 nm band with hollow-core photonic bandgap fibre as gas cell. Electron. Lett.

[4] Cox F.M., Argyros A., Large M.C.J., Kalluri S. (2007). Surface enhanced Raman scattering in a hollow core microstructured optical fiber. Opt. Express.

[5] Rindorf L., Hoiby P.E., Jensen J.B., Pedersen L.H., Bang O., Geschke O. (2006). Towards biochips using microstructured optical fiber sensors. Anal. Bioanal. Chem.

[6] Benabid F., Knight J.C., Russell P.S. (2002). Particle levitation and guidance in hollow-core photonic crystal fiber. Opt. Express.

[7] Amezcua-Correa R., Gèrôme F., Leon-Saval S.G., Broderick N.G.R., Birks T.A., Knight J.C. (2008). Control of surface modes in low losshollow-core photonic bandgap fibers. Opt. Express.

[8] Aref S.H., Amezcua-Correa R., Carvalho J.P., Frazao O., Caldas P., Santos J.L., Araujo F.M., Latifi H., Farahi F., Ferreira L.A. (2009). Modal interferometer based on hollow-core photonic crystal fiber for strain and temperature measurement. Opt. Express.

[9] Dong B., Hao E.J. (2011). Temperature-insensitive and intensity-modulated embedded photonic-crystal-fiber modal-interferometer-based microdisplacement sensor. J. Opt. Soc. Am. B.

[10] Zhang H., Liu B., Wang Z., Luo J.H., Wang S.X., Jia C.H., Ma X.R. (2010). Temperature-insensitive displacement sensor based on high-birefringence photonic crystal fiber loop mirror. Opt. Appl.

[11] Bravo M., Pinto A.M.R., Lopez-Amo M., Kobelke J., Schuster K. (2012). High precision micro-displacement fiber sensor through a suspended-core Sagnac interferometer. Opt. Lett.

[12] Kulkarni V.K., Lalasangi A.S., Pattanashetti I.I., Raikar U.S. (2006). Fiber optic micro-displacement sensor using coupler. J. Optoelectron. Adv. Mater.

[13] Han M., Zhang Y., Shen F.B., Pickrell G.R., Wang A.B. (2004). Signal-processing algorithm for white-light optical fiber extrinsic Fabry-Perot interferometric sensors. Opt. Lett.

